# Mechano-Biological Computer Model of Scaffold-Supported Bone Regeneration: Effect of Bone Graft and Scaffold Structure on Large Bone Defect Tissue Patterning

**DOI:** 10.3389/fbioe.2020.585799

**Published:** 2020-11-11

**Authors:** Camille Perier-Metz, Georg N. Duda, Sara Checa

**Affiliations:** ^1^Julius Wolff Institute, Charité-Universitätsmedizin, Berlin, Germany; ^2^MINES ParisTech – PSL Research University (Paris Sciences & Lettres), Paris, France; ^3^Berlin Institute of Health (BIH) Center for Regenerative Therapies, Charité-Universitätsmedizin, Berlin, Germany

**Keywords:** mechano-biology, bone defect healing, 3D-printed scaffold design, bone tissue engineering, tissue regeneration, bone graft

## Abstract

Large segmental bone defects represent a clinical challenge for which current treatment procedures have many drawbacks. 3D-printed scaffolds may help to support healing, but their design process relies mainly on trial and error due to a lack of understanding of which scaffold features support bone regeneration. The aim of this study was to investigate whether existing mechano-biological rules of bone regeneration can also explain scaffold-supported bone defect healing. In addition, we examined the distinct roles of bone grafting and scaffold structure on the regeneration process. To that end, scaffold-surface guided migration and tissue deposition as well as bone graft stimulatory effects were included in an *in silico* model and predictions were compared to *in vivo* data. We found graft osteoconductive properties and scaffold-surface guided extracellular matrix deposition to be essential features driving bone defect filling in a 3D-printed honeycomb titanium structure. This knowledge paves the way for the design of more effective 3D scaffold structures and their pre-clinical optimization, prior to their application in scaffold-based bone defect regeneration.

## Introduction

Large segmental bone defects represent a clinical challenge for which current treatment procedures (e.g., autologous bone grafting, Masquelet technique, BMP-2) present several drawbacks such as the need for an additional surgery, limited graft availability, donor site morbidity or side effects (Roddy et al., 2018). 3D-printed scaffolds are appealing alternatives due to their versatility in the design process and ease to customize to the patient-specific defect situation. In pre-clinical studies, several 3D-printed scaffolds have shown their potential to support bone defect healing (Cobos et al., 2000; Reichert et al., 2012; Lovati et al., 2016; Shah et al., 2016; Pobloth et al., 2018; Reznikov et al., 2019); however, their translation to the clinical setting remains challenging (Hollister, 2009; Hollister and Murphy, 2011). One of the reasons is a lack of understanding of how known influencing features of 3D-printed scaffold structure (e.g., pore size, geometry, stiffness, curvature, material) independently and together impact the biology of the regeneration process (Hollister, 2009; Metz et al., 2020). Therefore, current scaffold design processes are mainly based on trial and error approaches. An in-depth understanding of how structural design parameters impact each other and the biology of bone defect healing appears mandatory to allow predictive healing to occur. Specifically, a 3D-printed bone scaffold should not aim to replace the missing bone identically but rather to provide a suitable environment for bone to regrow. Computer modeling (benchmarked against *in vivo* data) could help unraveling the principles of bone defect regeneration and allow a pre-operative planning of a patient own scaffolding strategy and thus the optimization of a 3D-printed customized scaffold enabling bone defect healing.

*In silico* mechano-biological models have been previously extensively developed and validated against *in vivo* data for successful bone healing, using both continuous approaches (Lacroix and Prendergast, 2002; Isaksson et al., 2006, 2007; Burke and Kelly, 2012) and agent-based models (Byrne et al., 2011; Checa et al., 2011; Vetter et al., 2012; Repp et al., 2015; Borgiani et al., 2019). Some of these models have also been used to investigate scaffold-supported bone regeneration (Byrne et al., 2007; Sanz-Herrera et al., 2008; Checa and Prendergast, 2010; Sun et al., 2013; Liu et al., 2019) and the interaction between scaffold design properties and tissue regeneration for bone (Bashkuev et al., 2015; Boccaccio et al., 2016) or cartilage applications (Kelly and Prendergast, 2006). However, to the authors’ knowledge, their predictive capability has never been tested against experimental data. Moreover, most of these models have ignored or highly simplified any mechanical or biological interaction between the scaffold and the regeneration process.

Experimentally, several scaffold design features have been shown to play an essential role in bone ingrowth and defect regeneration, e.g., the scaffold’s material properties (Lichte et al., 2011), porosity or pore size (Bose et al., 2012). In addition, it has been suggested that scaffolds act as a template along which the bone regeneration process occurs (Cipitria et al., 2012; Pobloth et al., 2018). Specifically, it has been postulated that a scaffold surface can guide cell migration and tissue deposition processes (Kellomäki et al., 2000; Sengers et al., 2007; Cipitria et al., 2012; Werner et al., 2018), however, the relative role of scaffold guidance for bone regeneration remains largely unclear.

To identify key design features of 3D-printed scaffolds in defect regeneration is challenging since biomaterials are usually not used alone but in combination with bone grafting or BMP-2 to increase the success rate of the bone defect regeneration (Viateau et al., 2007; Pobloth et al., 2018). It is thought that cells (pre-osteoblasts and/or osteoprogenitors) contained in a bone graft contribute to healing and that bone grafts have osteoconductive (guiding new bone ingrowth) and osteoinductive (enhancing bone deposition) properties (Finkemeier, 2002), however, how these properties contribute to the overall healing process remains poorly understood. Until now, a systematic analysis of these bone graft stimulatory features is lacking, as well as the understanding of which of the 3D-printed scaffold features are essential in bone defect regeneration.

The aim of this study was (1) to investigate the potential of an existing bone regeneration mechano-biological computer model to explain scaffold-supported bone healing and (2) to investigate the relative contribution of scaffold guidance and bone graft stimulation to bone regeneration within a scaffold. To do this, new features were implemented in a mechano-biological *in silico* model for bone healing (Checa et al., 2011) to describe graft- and scaffold-supported bone regeneration and tested against *in vivo* data.

## Materials and Methods

### Simulated *in vivo* Experiments

A previously described *in vivo* study (Pobloth et al., 2018) was used to compare *in silico* predictions to *in vivo* observations of large bone defect tissue patterning. Details of the experimental study design are only briefly described here. A large tibial bone defect in sheep was used to study bone defect healing. To investigate the role of scaffold overall stiffness on bone regeneration, 12 sheep underwent a 4 cm osteotomy in the right tibia that was filled with a relatively soft or stiff customized 3D printed titanium scaffold. Scaffolds had a honeycomb structure, identical topology but different strut thicknesses (1.2 or 1.6 mm) leading to overall soft (0.84 GPa) or stiff (2.88 GPa) scaffolds. The osteotomy was held in place using a steel locking compression plate. In addition, autografts taken from the iliac crest were crushed and filled into the scaffold pores before implantation. Radiographs were performed every 4 weeks to evaluate progress in bone defect regeneration. The animals were sacrificed 24 weeks post-surgery, tibia was harvested and histomorphometrical analysis was performed on a mid-sagittal cut to visualize bone and cartilage formation within the defect. In this study, the soft scaffold was used to investigate the influence of scaffold guidance and bone graft stimulation on scaffold-supported bone regeneration. Therefore, all parameter analyses were done for the soft scaffold configuration. Only the model with best prediction capabilities was then tested in the stiff scaffold configuration.

### *In silico* Baseline Bone Regeneration Model

**FIGURE 1:**
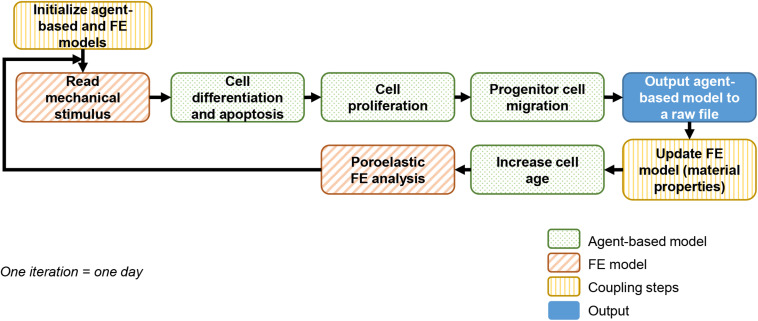
Bone regeneration computer model flowchart.

A previously described and experimentally validated bone regeneration computer model was used (Checa et al., 2011). The computer model couples agent-based and finite element (FE) models to simulate bone growth within the healing region as depicted in Figure 1. This framework will be referred to as “*baseline model*” in the following and described in sections “Agent-Based Model” and “Finite Element model.”

#### Agent-Based Model

An agent-based model accounting for cellular activities (proliferation, apoptosis, migration, and differentiation) was implemented in C++. The callus space was discretized into a 3D grid (spacing 100 μm) in which each point was occupied by maximum one of the following cell phenotypes: progenitor cell, fibroblast, chondrocyte, mature osteoblast or immature osteoblast. Since the distance between agents was larger than the expected cell size, a single agent would actually contain several cells as well as the corresponding extracellular matrix. Therefore, cells and tissues were identified in the model.

Unless specified differently, progenitor cells were initially seeded along the periosteum and in the marrow cavity, occupying 30% of all free positions. They were implemented to migrate randomly at a mean speed of 30 μm/h (Appeddu and Shur, 1994). Cell differentiation toward another phenotype was possible once the progenitor cell was mature (more than 6 days), depending on a mechano-regulation algorithm based on shear strain and fluid velocity (Huiskes et al., 1997; Prendergast et al., 1997) with threshold values following Lacroix and Prendergast (Lacroix and Prendergast, 2002; Table 1). Cells and corresponding tissues being identified, the simulated differentiation included the matrix deposition process.

**TABLE 1:** Mechano-regulation algorithms for progenitor cell differentiation.

Stimulus: $S=\frac{\gamma }{\text{a}}+\frac{\text{v}}{\text{b}}\,\left[c\,p\,s\,b\,r\,e\,a\,k\right]\,\gamma$: shear strain, v: fluid velocity *a* = 0.0375^a^, *b* = 0.003 mm/s^a^	Bone resorption	Mature bone	Immature bone	Cartilage	Fibrous tissue
Thresholds^a,b^	S ≤ 0.01	0.01 < S ≤ 0.53	0.53 < S ≤ 1	1 < S ≤ 3	3 < S

*^*a*^(Huiskes et al., 1997). ^*b*^(Lacroix and Prendergast, 2002).*

All cell phenotypes were allowed to proliferate providing that their surrounding mechanical microenvironment was appropriate (depending on the mechano-regulation thresholds) or would undergo apoptosis otherwise, with proliferation and apoptosis rates given in Table 2.

**TABLE 2:** Cell activity rates (adapted from Checa et al. (2011).

Cell type	Proliferation rate (/day)	Apoptosis rate (/day)	Differentiation rate (/day)	Migration speed (μm/h)
Progenitor cells	0.6^a^	0.05^a^	0.3^a,1^	30^b^
Fibroblasts	0.55^a^	0.05^a^	–	–
Chondrocytes	0.2^a^	0.1^a^	–	–
Osteoblasts	0.3^a^	0.16^a^	–	–

*^1^Unless specified differently. ^*a*^(Isaksson et al., 2008). ^*b*^(Appeddu and Shur, 1994).*

#### Finite Element Model

**FIGURE 2:**
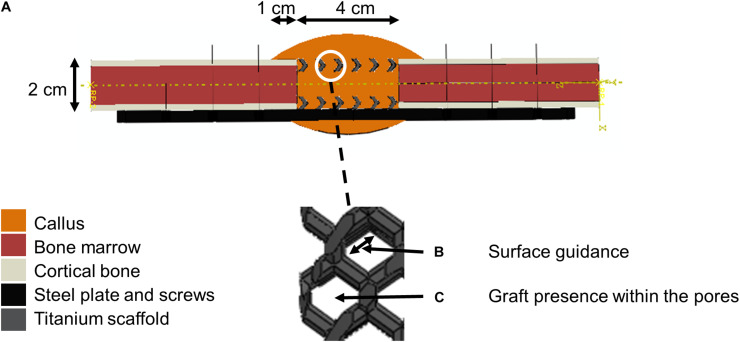
Model setup description. **(A)** Finite element model of the 4 cm osteotomy with a zoom within scaffold pores. The color code given on the left shows the different materials defined at the initial time point (after implantation). **(B)** Scaffold-surface guidance effects. **(C)** Bone graft stimulatory effects.

A biphasic poroelastic finite element (FE) model of the 4 cm tibial osteotomy was developed in ABAQUS/CAE v.6.12 (Simulia, Rhode Island) based on an idealized geometry: intact bone extremities were modeled as hollow cylinders (radius 10 mm) representing the cortical bone shell (2.5 mm thick) and containing the bone marrow cavity (radius 7.5 mm). The callus was obtained by rotating a circle arc of maximum thickness 10 mm at mid-height, and overlapping 10 mm over intact cortical bone extremities. The scaffold geometries were imported from original CAD files of the scaffolds used in the experimental setup. The steel fixation plate was also imported from a CAD file while the eight steel screws were approximated as beam elements with circular section (Figure 2A).

All biological tissues were modeled as poroelastic materials with properties given in Table 3. Titanium and steel were considered linear elastic materials, with Young’s modulus 104 and 210 GPa, respectively, and Poisson’s ratio 0.3.

**TABLE 3:** Tissue material properties (adapted from Checa et al., 2011).

	Granulation tissue	Fibrous tissue	Cartilage	Immature bone	Mature bone	Cortical bone	Marrow
Young’s modulus (MPa)	0.2	2	10	1,000	17,000	17,000	2
Permeability (10^–14^ s.m^4^/N)	1	1	0.5	10	37	0.001	1
Poisson’s ratio	0.167	0.167	0.3	0.3	0.3	0.3	0.167
Bulk modulus grain (MPa)	2,300	2,300	3,700	13,940	13,940	13,920	2,300
Bulk modulus fluid (MPa)	2,300	2,300	2,300	2,300	2,300	2,300	2,300

Mechanical loading conditions aimed to simulate the peak load under normal walking conditions: a proximal-distal axial load (compression) of 1,372 N [corresponding to 2 body weights (BW)] and an anterior-posterior moment (bending) of 17.125 Nm (corresponding to 0.025 BWm at the fixed end of an intact bone) (Duda et al., 1997) were applied on the bone proximal extremity, the distal being constrained in rotation and translation (encastre). Pore pressure was constrained to be zero on the poroelastic materials’ outer surface (callus, cortical bone and marrow). The screws were fixed to the plate using multi-point constraints of type beam. Tie constraints were defined between callus and intact bone extremities, implant and callus, and intact bone and screws.

The model was meshed using second-order elements of the following types: hexahedral elements of average size 2.5 mm for the cortical bone, the bone marrow and the plate; beam elements of size 1 mm for the screws; and tetrahedral elements of average size 0.7 mm for the callus and the scaffold.

The callus was initially filled with granulation tissue. The FE model was updated iteratively to account for extracellular matrix (ECM) deposition and tissue mechanical property changes in the callus following a rule of mixtures (Lacroix et al., 2002): the mechanical properties of the different cell types present in an element of the FE model were averaged to compute the material properties of the element (Table 3). To account for the delay in actual ECM deposition, each element’s properties were averaged over the last 10 iterations, namely 10 days (Lacroix and Prendergast, 2002). The FE model was next run in ABAQUS/Standard v.6.12 (Simulia, Rhode Island) to compute the corresponding mechano-regulation stimulus.

### Simulation of the Bone Graft Stimulatory Effects

**FIGURE 3:**
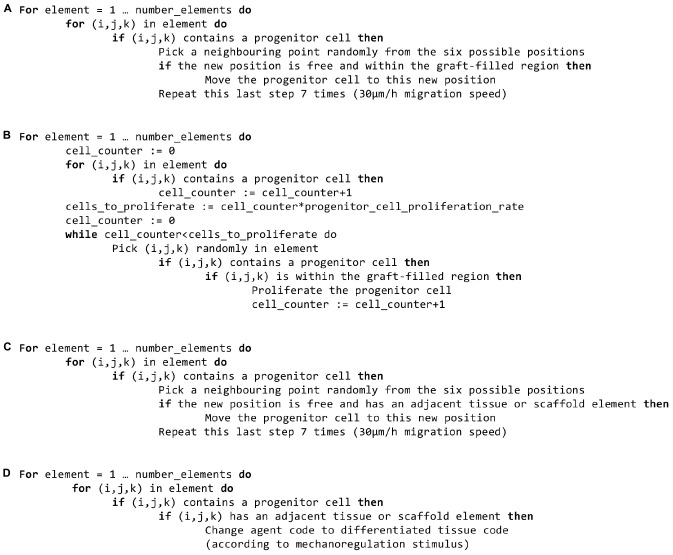
Pseudo-code for the newly implemented features. **(A)** Graft stimulatory effect on progenitor cell migration. **(B)** Graft stimulatory effect on progenitor cell proliferation. **(C)** Surface-guided migration. **(D)** Surface-guided extracellular matrix deposition.

The baseline bone healing algorithm was modified to account for three potential biological stimulatory effects of the bone graft present in the scaffold pores (Figures 2C, 3A,B and Table 4):

**TABLE 4:** Summary of the bone healing simulations and their characteristics.

Simulation name	Scaffold configuration	Figure	Latency period	Graft stimulatory effects	Surface guidance features
Baseline model	Soft	5D 4B (2)*	No	None	None
Non-stimulated bone regeneration model	Soft	5E	Yes	None	None
Graft stimulatory effect on cell migration	Soft	6A 4B (3)*	Yes	Osteoconductive	None
Graft stimulatory effect on cell proliferation	Soft	6B 4B (4)*	Yes	Osteoconductive	None
Graft stimulatory effect on cell differentiation	Soft	6C	Yes	Osteoinductive	None
Combined graft osteoconductive and osteoinductive effects	Soft	6D 4B (5)*	Yes	Osteoconductive + osteoinductive	None
Bone graft-contained progenitor cells	Soft	6E 4B (6)*	Yes	Initial seeding of progenitor cells	None
Combined bone graft-contained progenitor cells with osteoconductive and osteoinductive effects	Soft	6F 4B (7)*	Yes	Osteoconductive + osteoinductive + initial seeding of progenitor cells	None
Surface-guided migration with non-stimulated bone regeneration	Soft	7A	Yes	None	Surface-guided migration
Surface ECM deposition with non-stimulated bone regeneration	Soft	7B	Yes	None	Surface ECM deposition
Surface-guided migration with bone graft osteoconductive effects	Soft	7C	Yes	Osteoconductive	Surface-guided migration
Surface ECM deposition with bone graft osteoconductive effects	Soft	7D 4B (8)*	Yes	Osteoconductive	Surface ECM deposition
Surface ECM deposition with bone graft osteoconductive effects	Stiff	8B	Yes	Osteoconductive	Surface ECM deposition

**FIGURE 4:**
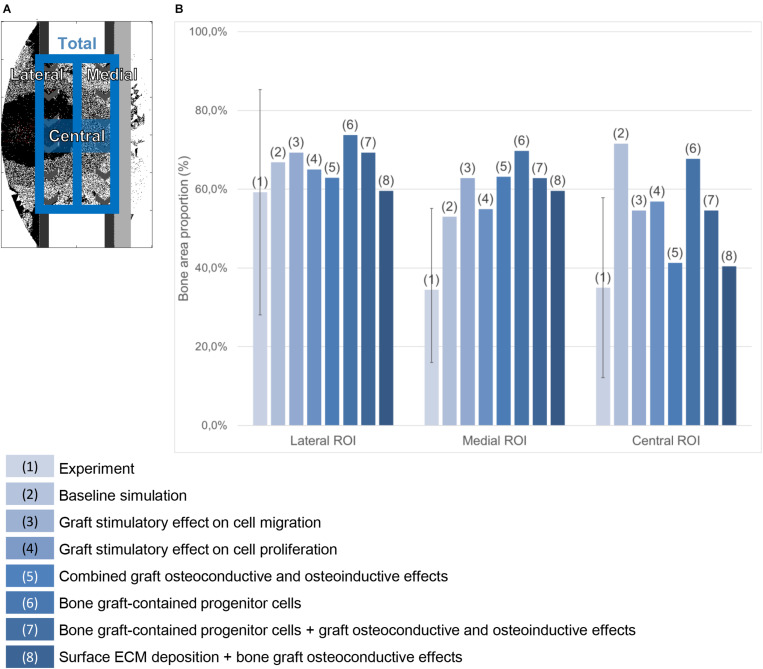
Bone area quantification in the mid-sagittal plane. **(A)** Regions of interest. **(B)** Percentage quantification in the experiment (the error bar shows the minimal and maximal values among the six animals) and in various simulation cases referred to in the legend on the bottom.

*The number in parentheses refers to the bar chart reference number for the simulation case on Figure 4B.

1. *Osteoconductive effects* (guiding new bone ingrowth) (Finkemeier, 2002) were modeled by limiting progenitor cell migration and/or proliferation after a latency period (15 days) to the regions containing graft. This latency period was defined to account for the fact that progenitor cells would stop being biologically active after a few days without a biological stimulus.
2. *Osteoinductive effects* (enhancing bone deposition) (Finkemeier, 2002) were modeled by increasing the rate of progenitor cell differentiation toward osteoblasts from 0.3 to 0.5 in the regions containing graft. In this case, progenitor cell migration and proliferation were limited to the latency period defined above.
3. *Bone graft-contained progenitor cells* (Finkemeier, 2002) were modeled by the initial seeding of progenitor cells in 0.1% of the available graft volume. A 15-days latency period was also implemented in this case.

Those effects were investigated both independently and in the following combinations: osteoconductive and osteoinductive effects without and with bone graft-contained progenitor cells.

Additionally, a hypothetical case corresponding to the absence of any stimulatory effect of the graft was simulated by implementing a latency period in the baseline simulation, after which both progenitor cell migration and proliferation would stop. This “non-stimulated bone regeneration model” served as a new baseline to investigate further effects.

### Simulation of Scaffold-Surface Guidance

Two features were implemented to investigate the role of scaffold-surface guidance (Sengers et al., 2007; Cipitria et al., 2012; Werner et al., 2018) on bone regeneration (Figures 2B, 3C,D and Table 4):

1. *Surface-guided migration*: a progenitor cell was allowed to migrate to a randomly picked new position only if at least one of the new position’s neighboring points was occupied by tissue (bone, cartilage, fibrous tissue) or scaffold. This assumes that a progenitor cell can only migrate along an existing tissue or scaffold-surface and not within the granulation tissue.
2. *Surface-guided extracellular matrix (ECM) deposition*: a progenitor cell was allowed to differentiate into a new phenotype and thus deposit the corresponding tissue only if at least one of its neighboring positions was occupied by tissue or scaffold. This assumes that new tissue deposition cannot happen within granulation tissue but needs a substrate (pre-existing tissue, scaffold) to attach to.

These two features were compared to the non-stimulated bone regeneration model (as explained in section “**Simulation of the Bone Graft Stimulatory Effects”**) and in combination with graft osteoconductive effects (section “**Simulation of the Bone Graft Stimulatory Effects”**). Since *in vivo* tissue formation was not observed along the plate (Pobloth et al., 2018), the plate was not simulated to provide any guidance in any of the cases.

### Output Analysis

Computer model predictions were compared to X-ray and histological data. Therefore, algorithms were developed to extract similar images as computer model output. In addition, quantification of the regenerated bone was compared to histological measures.

#### Output for X-ray-Like Image Prediction

To compare the time evolution of the bone healing process within the scaffolds, X-ray-like images were computationally generated at the same time points than experimentally: 0, 4, 8, 12, 16, 20, and 24 weeks. Neglecting the surrounding soft tissues and their X-ray scattering, the images were obtained using the Beer-Lambert law:

$$\begin{array}{c} I=I_{0}\,e^{-k\,x} \end{array}$$

with I the observed intensity, I0 the initial intensity (an arbitrary value 1 was chosen for normalization), k the material attenuation coefficient and x the material thickness (Bushberg, 2012). Taking the grid spacing (100 μm) of the agent-based model for x and the attenuation coefficients defined by the NIST,^1^ the attenuation over all crossed grid points in the X-ray beam direction was summed, thereby revealing the corresponding radiograph. The contrast was then adapted to be as close as possible to the experimental images.

#### Output for Histological-Like Image Prediction

Histology-like images were extracted from the computer model predictions to compare with the experimental histology images obtained at 24 weeks post-surgery using Safranin Orange/von Kossa staining. To do so, the predicted tissue distribution in the mid-sagittal plane was represented with 100 μm-sided pixels in colors similar to the staining: black for bone, dark red for cartilage and light red for fibrous tissue. Zones without any tissue were left white, while intact bone, scaffold and fixation plate were depicted in gray nuances. To allow comparison between different *in silico* predictions, the images were generated at the same time points as the X-ray pictures: 4–24 weeks, with a 4-week interval.

#### Quantification of Predicted Bone Tissue Area in the Mid-Sagittal Plane

To allow for a quantitative comparison, the relative bone tissue area on the mid-sagittal plane was evaluated after 24 weeks. The experimental histology pictures were segmented for bone (in black) using ImageJ (Abramoff et al., 2003), after closing the pores (as they are not predicted by the bone regeneration computer model presented here). In the predicted histology-like images of bone healing, the lattice points of the mid-sagittal plane occupied by bone were counted at time point 24 weeks. This quantification was conducted only for the simulation cases showing a prediction qualitatively close to the experimental pictures, e.g., with a completely healed defect after 24 weeks. The quantifications are given as a percentage of the available surface in three different regions of interest (lateral, medial and central) described in Figure 4A.

## Results

The output obtained for the *baseline model* and the *non-stimulated bone regeneration model* were compared to the experimental data (section “Baseline Simulation”). Then, the results of the addition of *bone graft stimulatory effects* and *scaffold-surface guidance features* were investigated individually and in combination to compare them between each other and to the experimental data (as summarized in Table 4).

### Baseline Simulation

**FIGURE 5:**
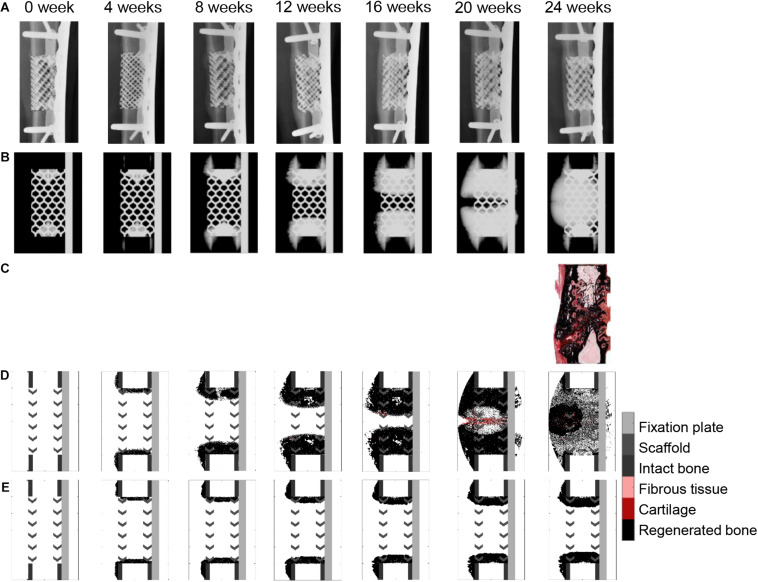
Bone healing dynamics over time: **(A)** *in vivo*, X-ray images (anteroposterior); **(B)** *in silico*, simulated X-ray images (anteroposterior) using the baseline bone healing algorithm (Checa et al., 2011); **(C)** *in vivo*, 24-week histology image (mid-sagittal), Safranin Orange/von Kossa staining; **(D)** *in silico*, simulated histology pictures (mid-sagittal) with baseline simulation; **(E)** *in silico*, simulated histology pictures (mid-sagittal) in the non-stimulated bone regeneration model. The color code for the simulated histology pictures is given on the right.

When a previously validated bone healing algorithm for uneventful bone healing (Checa et al., 2011) was used, the bone defect regeneration across a large defect (4 cm) filled with a titanium scaffold (Pobloth et al., 2018) could not be predicted. The experimentally observed bone tissue formation patterns in the defect were neither reproduced in their dynamics nor by comparing the end time point (24 weeks) (Figures 5A–D and Supplementary Figure S1). The baseline bone healing algorithm predicted large amounts of regenerated bone that occupied most of the callus volume and capped the bone marrow cavities proximally and distally. However, *in vivo* an opening of the bone marrow cavity was observed and bone formation occurred mainly within the scaffold pores. A smaller amount of bone was observed *in vivo* on the medial side (under the plate) while more bone formation bridging the defect was observed on the lateral side (Figure 4B). This difference was not detected by the *in silico* predictions. Furthermore, the amount of bone in the central region of interest was largely overestimated: 72% instead of 35% (Figure 4B). *In vivo*, tissue formation was characterized by fibrocartilage patterning or endochondral ossification along the scaffold struts that was hardly predicted by the bone healing algorithm *in silico*. In addition, the bone healing algorithm predicted a dynamic filling process with bridging of the defect after roughly 18 weeks, whereas experimentally bridging occurred on average after 12 weeks. After 18 weeks, the computer model algorithm predicted significant bone remodeling that was not observed *in vivo*.

If the stimulatory effects were taken out in the baseline bone healing algorithm (non-stimulated bone regeneration model: progenitor cell migration and proliferation stopped after a 15-days latency period; Figure 5E), healing was largely impaired, resulting in non-union and capping of the bone marrow cavities.

### Bone Graft Stimulatory Effects in Scaffold-Supported Bone Regeneration

**FIGURE 6:**
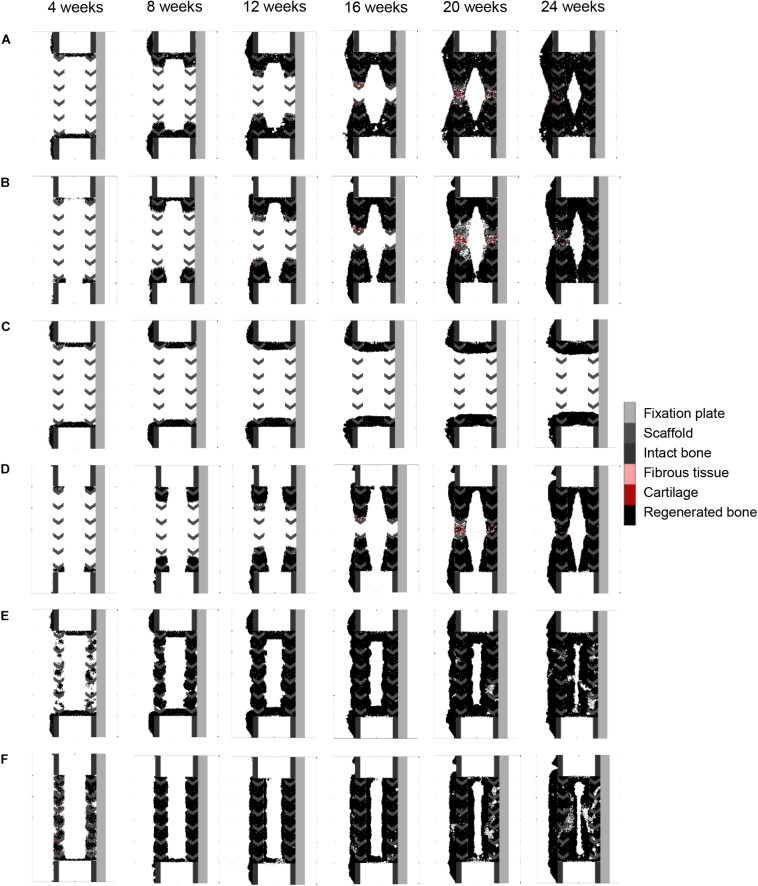
Bone graft stimulatory effects on scaffold-supported bone regeneration: **(A)** graft stimulatory effect on cell migration (osteoconductivity); **(B)** graft stimulatory effect on cell proliferation (osteoconductivity); **(C)** graft stimulatory effect on cell differentiation; **(D)** combined graft osteoconductive and osteoinductive effects; **(E)** bone graft-contained progenitor cells; **(F)** combined bone graft-contained progenitor cells with osteoconductive and osteoinductive effects. The color code is given on the right.

Making graft presence a prerequisite for progenitor cell migration or proliferation (bone graft osteoconductive effects: Figures 6A,B) resulted in bone growth patterning confined within the scaffold pores. However, it did not reproduce the experimentally observed differences in bone regeneration between medial (under the plate) and lateral sides (Figure 4B). The dynamics of the simulated osteoconductive effects was slightly slower than the baseline simulation (bridging achieved after ca. 20 weeks), and consequently slower than *in vivo* (average bridging after 12 weeks).

Enhancing progenitor cell differentiation into osteoblasts in regions containing graft (bone graft osteoinductive effects: Figure 6C) did not have much impact when compared to the non-stimulated bone regeneration model, leading to similar patterning and very slow regeneration process.

When combining bone graft osteoconductive and osteoinductive effects (Figure 6D), the patterning resulting from isolated osteoconductive effects was further enhanced, eventually confining bone deposition within the scaffold pores and achieving bridging in ca. 20 weeks. In particular, the central ROI quantification fitted well the experimental data: 41 vs. 35% (Figure 4B). However, not only did the final patterning not reproduce the medial-lateral difference observed *in vivo*, but it also led to a marrow cavity capping by regenerated bone consistently not seen *in vivo*.

Modeling the filling of the scaffold with bone grafting as an additional source of progenitor cells (Figure 6E) resulted in a much faster process, bridging being predicted after 10 weeks. Different from the experimental results was the very homogeneous growing process, bone being predicted across the whole defect length simultaneously instead of growing from the bone osteotomy cuts as observed *in vivo* on X-ray images (Figure 5B). Besides, bone was overpredicted in the medial and central ROIs (Figure 4B). If the bone healing algorithm included all three graft stimulatory affects (bone graft-contained progenitor cells, osteoconductive and osteoinductive properties) (Figure 6F), bone bridging was predicted after 9 weeks. In addition, the bone marrow channel opened, and a trend to more bone formation on the lateral side compared to the medial side (under the plate) could be observed: 69 and 63% instead of 59 and 34% in the simulated and experimental images, respectively (Figure 4B). The central ROI prediction of 55% was within the range of the experimental measures (maximal value of 58%). However, the healing process was very homogeneous through the entire defect instead of starting from the bone osteotomy cuts.

### Scaffold-Surface Guidance Features in Scaffold-Supported Bone Regeneration

**FIGURE 7:**
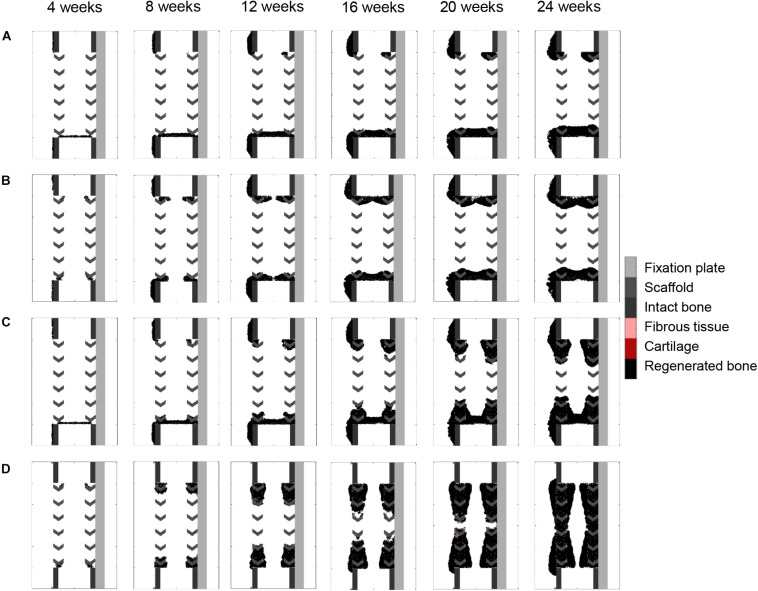
Surface guidance features: **(A)** surface-guided migration with non-stimulated bone regeneration; **(B)** surface ECM deposition with non-stimulated bone regeneration; **(C)** surface-guided migration with bone graft osteoconductive effects; **(D)** surface ECM deposition with bone graft osteoconductive effects. The color code is given on the right.

Surface guidance features further slowed down the impaired regeneration predicted in the non-stimulated bone regeneration model, confining bone growth mostly to the scaffold pores (Figures 7A,B). Both cases resulted in a non-union, and marrow cavity capping was observed due to osteoblast proliferation and consequent bone deposition.

Combining surface guidance features with bone graft osteoconductive effects (Figures 7C,D and Supplementary Figure S1) resulted in a better patterning at the end of the regeneration process, with bone being confined to the scaffold pores. Thus, the central ROI quantification was close to the experimental measures: 40 vs. 34% (Figure 4B). However, there was hardly no difference between medial and lateral bone volumes (60% bone area in both cases), contrary to *in vivo* (Figure 4B). The surface ECM deposition feature (Figure 7D) resulted in a slightly slower process than the baseline simulation (bridging in 22 weeks), while the surface-guided migration (Figure 7C) predicted non-union after 24 weeks, what did not match the *in vivo* observations.

### Surface ECM Deposition and Bone Graft Osteoconductive Effects in the Stiff Scaffold

**FIGURE 8:**
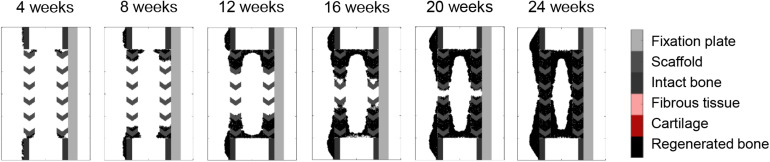
Stiff scaffold (strut width 1.6 mm)—surface ECM deposition with bone graft osteoconductive effects.

When applying the simulation setup with graft osteoconductive effects and surface ECM deposition to the stiff scaffold design (Figure 8), the regeneration process was very similar to the soft scaffold design (Figure 7D). It did not show the differences observed *in vivo*, namely a significantly slower process (bridging in at least 24 weeks) and less regenerated bone. In particular, when quantifying the bone proportion in the ROIs defined in Figure 4, proportions of 57, 56, and 36% were predicted by the model in the lateral, medial and central ROIs, respectively, while experimentally they were in average of 44, 25, and 21%.

## Discussion

3D printed scaffolds appear as a promising alternative for the treatment of large bone defects; however, until now there is little understanding of the mechano-biological rules driving scaffold-supported bone regeneration. In this study, we analyzed whether a previously validated bone healing algorithm could predict scaffold-supported bone defect healing to identify specific mechano-biological factors which might differ between these modes of bone regeneration. The new bone defect healing algorithm introduced in the present work revealed specific mechanisms behind scaffold-supported bone healing. Our findings illustrate the relevance of (1) scaffold surfaces as a guide for bone defect regeneration and (2) the stimulatory role of autologous bone grafting as filling for such scaffolds. Both features taken together allowed to mimic the experimentally observed bone tissue patterning in a large bone defect supported by a titanium scaffold and autologous bone grafting, both qualitatively and quantitatively.

Although already used to predict bone regeneration within scaffolds (Sanz-Herrera et al., 2008; Checa and Prendergast, 2010; Boccaccio et al., 2016), most of the existing mechano-biological computer models had never been validated against experimental data. In this study, we implemented an existing computer model of bone regeneration which had been validated for uneventful bone healing (Lacroix and Prendergast, 2002; Checa et al., 2011) and tested its potential to predict bone regeneration within a large bone defect in sheep supported with a titanium scaffold (Pobloth et al., 2018). Our results show that mechano-biological models of uneventful bone regeneration are not able to explain scaffold-supported bone regeneration. For the specific experimental setup investigated in this study, our simulations showed an overestimation of the bone formation, with different dynamics and patterning from those observed experimentally.

Based on experimental observations of limited bone formation in untreated large bone defects (Mehta et al., 2012), an activity latency period (15 days) for progenitor cells was implemented in the baseline model to simulate a hypothetical case of non-stimulated bone regeneration. Simulation results led to non-union with bone marrow cavity capping, as observed in experimental studies on non-unions (Mehta et al., 2011; Schlundt et al., 2018). This approach has been already used to model impaired healing *in silico* (Borgiani, 2020).

In this study, two novel features were introduced in a pre-existing bone healing algorithm: the bone graft stimulatory effects and the scaffold-surface guidance features. Although the exact composition and effect of autologous bone grafts are not well known and described yet, it has been hypothesized that they are an additional source of progenitor cells (direct effect on bone formation) and present osteoconductive and osteoinductive properties (indirect effect on bone formation), thus guiding the regeneration process and enhancing bone deposition (Finkemeier, 2002). To the authors’ knowledge, the relative contribution of those potential effects has not been investigated yet with the help of *in silico* modeling. We found here that bone graft-contained progenitor cells would lead to a bone healing process different to the one observed *in vivo*: predictions showed concurrent bone growth throughout the entire defect at the same pace and not from the intact bone extremities, as seen in X-ray images. Additional graft stimulatory effects might be related to the presence of proteins enhancing the recruitment and activity levels of the progenitor cells. Thus, the osteoconduction of the graft was described by a limitation of progenitor cell migration and proliferation to regions containing graft, what led to a regeneration process confined within the scaffold pores. When combining osteoinductive (progenitor cell enhanced differentiation into bone) and osteoconductive effects with graft-contained progenitor cells, a good agreement between *in silico* and *in vivo* results in terms of final bone patterning was achieved, however, the dynamics of the healing did not reproduce experimental findings with bridging starting from the intact extremities. To the author’s knowledge, only one other group included graft features within an *in silico* model for bone regeneration: Sanz-Herrera et al. (2008) accounted for enhanced osteoconduction in a grafted scaffold *via* a better cell adhesion efficiency. Here, our results suggest that bone graft osteoconductive effects play a major role for the regeneration of the bone, while graft-contained progenitor cells do not seem major actors in this healing process.

Second, scaffold-surface guidance effects on bone regeneration were investigated, assuming that the scaffold provides a template on which tissue is preferentially deposited and/or along which progenitor cells preferentially migrate. Structured scaffold surface was indeed shown to guide the bone regeneration process in experimental studies (Cipitria et al., 2012; Berner et al., 2014; Pobloth et al., 2018). This is however dependent on the type of scaffold, coating and surgical approach: Reichert et al. (2011), for instance, reported a bone regeneration process happening mostly from the endosteal side and within the bone marrow. Here, using surface ECM deposition in addition to the bone graft osteoconductive effects resulted in bone healing predictions that were closer to the experimental data. Only few computer models have previously investigated the effect of scaffold guidance on bone regeneration. Schmitt et al. compared computer model predictions with histological images of mandible regeneration to study bone ingrowth within a titanium scaffold (Schmitt et al., 2016). They obtained good correlation in the bone patterning within the scaffold pores using a diffusion-based setup coupled with a mechanical framework (principal stresses). However, they took only progenitor cells and ossification into account (without including any other phenotype) and restricted the study to 2D. The isolated effect of scaffold guidance (without bone graft) was also investigated by Paris et al. (2017). They developed a computer model to predict soft and mineralized tissue formation within a PCL 3D-printed scaffold as guided by scaffold surface curvature. They showed that curvature could account for the collagen and mineralized bone tissue patterning observed *ex vivo*. However, the model only investigated non-healing sample groups. In this study, using a computer model of bone regeneration, we investigated the mechanisms behind scaffold-supported bone healing.

Although our model was able to predict bone tissue formation within one of the scaffold designs investigated *in vivo* (the soft one) when implementing a combination of bone graft osteoconductive effects and surface ECM deposition, the same model failed to reproduce the experimental observations for the stiff scaffold configuration. Computer model predictions of bone healing within the stiff scaffold did not show good agreement with experimental observations, predicting neither less bone than in the soft one, nor the significantly slower dynamics. A reason for that could be that the stiff scaffold has a bigger surface area (ca. 1,000 mm^2^ vs. 840 mm^2^), so that the surface guidance features of our model may favor the healing in its presence. This suggests that the stiffer mechanical environment might have stronger effects not included in the current model, such as a mechanically driven influence on cellular migration (Lo et al., 2000; Von Offenberg Sweeney et al., 2005; Dietrich et al., 2018) or proliferation (Hannafin et al., 2006; Saha et al., 2006; Moreo et al., 2008). Further work is required to investigate which of those mechanisms can explain the *in vivo* differences.

Our approach of modeling bone defect healing has also limitations and does not explain all experimental observations. First, there was no or very limited bone growth under the fixation plate (medial side) experimentally (Pobloth et al., 2018), which was not predicted so extensively *in silico*. One reason for the differential growth might be the lower strains in this region leading to strain shielding. However, the differences in the predicted mechanical signals between the medial and lateral sides were not enough to lead to predictions of different bone healing patterns in both sides, suggesting that there might be additional effects not taken into account, such as a biological reaction to the plate or disrupted soft tissues due to the surgery. Second, most of the animals showed a fibrocartilage layer around the scaffold struts that was hardly seen *in silico*. This phenomenon could also be explained by mechanics, because higher strains directly around the scaffold struts would result in a fibrocartilage favorable stimulus. Those might not be captured in our model due to a too coarse finite element mesh. In addition, a biological reaction to the surface might also account for the lack of bone direct attachment, known to be highly dependent on the surface chemical treatment (Soares et al., 2018; Su et al., 2018; Pippenger et al., 2019) and topography (Davies et al., 2013; Liddell et al., 2017). Lastly, some limitations of the model should be mentioned: revascularization was not included in the model as elsewhere (Checa and Prendergast, 2010; Burke and Kelly, 2012) but was assumed to be sufficient not to slow down the regeneration process. Vascularization of scaffolds for large bone defects is generally limited and remains a key challenge in bone tissue engineering (Laschke et al., 2006; Bose et al., 2012; Stratton et al., 2016; Bienert, 2019), what could partially contribute to observed healing patterns. Future studies should further investigate the relative effect of angiogenesis on scaffold-supported healing. Lastly, a strong remodeling was predicted *in silico* in the baseline model due to the high sensitivity of the model to slight strain changes around the mature bone formation to resorption limit. This is however not likely to happen *in vivo*. Future *in silico* models should investigate the mechanical regulation of bone remodeling during healing.

To summarize, here we presented new model features for a coupled multiscale mechano-biological model for bone regeneration to predict scaffold-supported bone regeneration, including bone graft stimulatory effects (osteoconduction, osteoinduction, source of progenitor cells) and scaffold-surface guidance features. We showed that a combination of bone graft osteoconductive effects and scaffold-surface guided ECM deposition could explain the patterning and healing dynamics observed in an *in vivo* scaffold-supported large bone defect regeneration study. Validated *in silico* models of scaffold-supported bone regeneration are a powerful tool for the prediction of the healing outcome of a given large bone defect with a specific scaffold. Rather than mimicking intact bone mechanical properties and internal structure, scaffold design should make use of such *in silico* modeling approaches allowing for a yet missing *a priori* evaluation of scaffold performance in enhancing the natural bone regeneration process.

## Data Availability Statement

The raw data supporting the conclusions of this article will be made available by the authors, without undue reservation, to any qualified researcher. The computer model source files were made available on the following repository: https://github.com/camille-PM/mechanobio_bone.

## Author Contributions

SC and GD designed the study. CP-M developed *in silico* models and collected the data. CP-M and SC interpreted the data and drafted the manuscript. All authors read and revised the manuscript and approved its content.

## Conflict of Interest

The authors declare that the research was conducted in the absence of any commercial or financial relationships that could be construed as a potential conflict of interest.

## Footnotes

1 https://physics.nist.gov/PhysRefData/XrayMassCoef/tab4.html
