# Supplementary material for: Mechano-Biological Computer Model of Scaffold-Supported Bone Regeneration: Effect of Bone Graft and Scaffold Structure on Large Bone Defect Tissue Patterning
Source: Front Bioeng Biotechnol. 2020 Nov 11;8:585799. doi: 10.3389/fbioe.2020.585799 (PMC7686036; doi:10.3389/fbioe.2020.585799)
Supplement: Supplementary file 1 [file Image_1.PDF]

Supplementary Material

(A) (B)

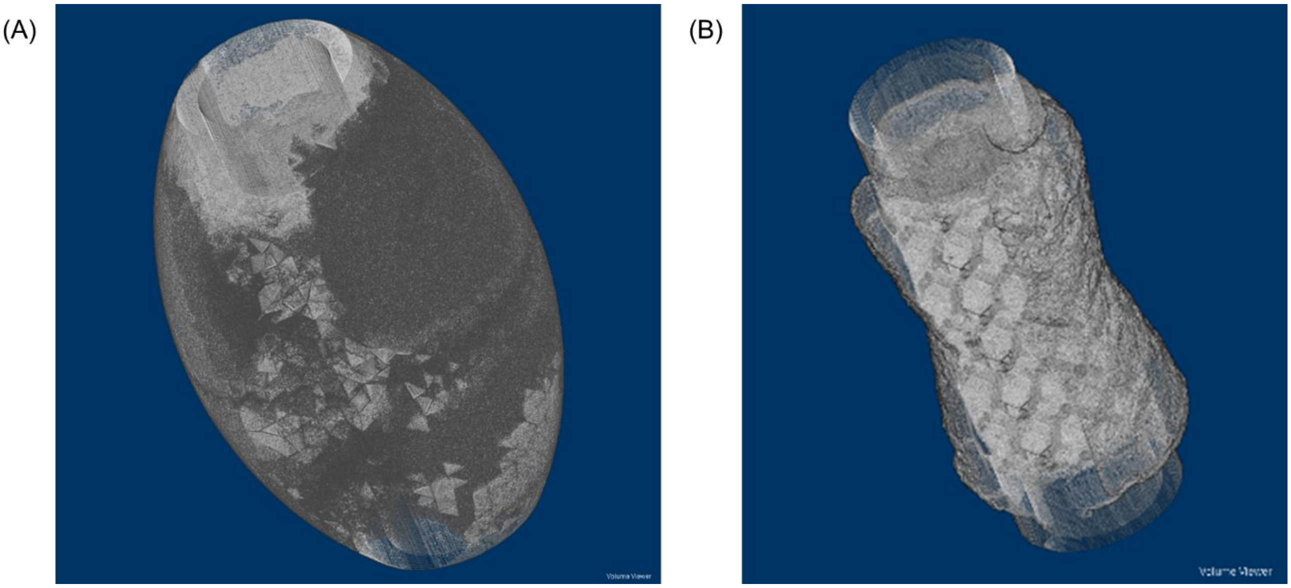

Supplementary Figure 1. MicroCT-like images of predicted regenerated bone after 24 weeks: (A) baseline simulation; (B) surface-guided ECM deposition with graft osteoconductive effects. The grey colour represents mature bone (the lighter the color, the denser the bone).
